# Supplementary material for: Biological Activities of the Fruit Essential Oil, Fruit, and Root Extracts of Ferula drudeana Korovin, the Putative Anatolian Ecotype of the Silphion Plant
Source: Plants (Basel). 2023 Feb 13;12(4):830. doi: 10.3390/plants12040830 (PMC9959981; doi:10.3390/plants12040830)
Supplement: Supplementary file 1 [file plants-12-00830-s001.zip › plants-2168387-supplementary.pdf]

Supplementary Material

Biological Activities of the Fruit Essential Oil, Fruit, and Root Extracts of *Ferula drudeana* Korovin, the Putative Anatolian Ecotype of the Silphion Plant †

Fatma Tosun [1,*], Fatih Göger [2], Gökalp İşcan [3], Mine Kürkçüoğlu [3], Fadıl Kaan Kuran [4] and Mahmut Miski [4,*]

[1] Department of Pharmacognosy, School of Pharmacy, İstanbul Medipol University, İstanbul 34083, Turkey
[2] Department of Pharmaceutical Botany, Faculty of Pharmacy, Afyonkarahisar Health Sciences University, Afyonkarahisar 03030, Turkey; fatih.goger@afsu.edu.tr
[3] Department of Pharmacognosy, Faculty of Pharmacy, Anadolu University, Eskişehir 26470, Turkey; giscan@anadolu.edu.tr (G.İ.); mkurkcuo@anadolu.edu.tr (M.K.)
[4] Department of Pharmacognosy, Faculty of Pharmacy, İstanbul University, İstanbul 34116, Turkey;
kaankuran@gmail.com
* Correspondence: ftosun@medipol.edu.tr (F.T.); mahmut.miski@gmail.com (M.M.); Tel.: +90-5334798035 (F.T.); +90-5455504455 (M.M.)
† Dedicate this publication to the victims of the February 6th South-East Anatolia Earthquake.

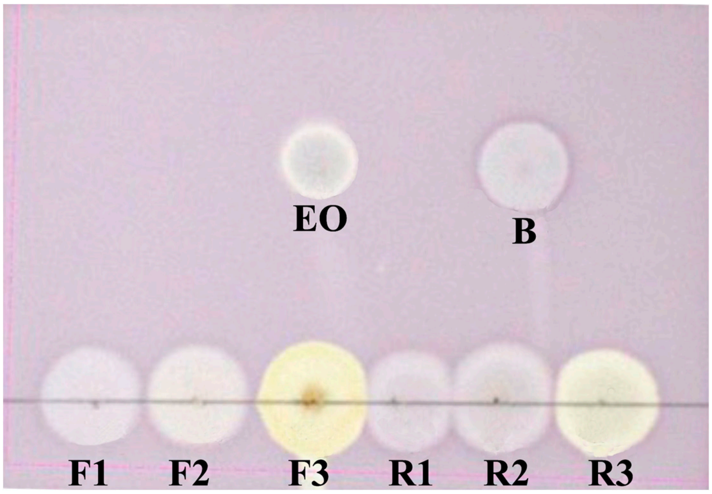

Figure. S1. The DPPH$^{\bullet}$ reagent evaluation of the essential oil and extracts of *Ferula drudeana*. **EO:** Essential oil, **B:** Blank, **F:** Fruit extracts, **R:** Root extracts (1: Petroleum ether, 2: Methylene chloride, 3: Methanol)

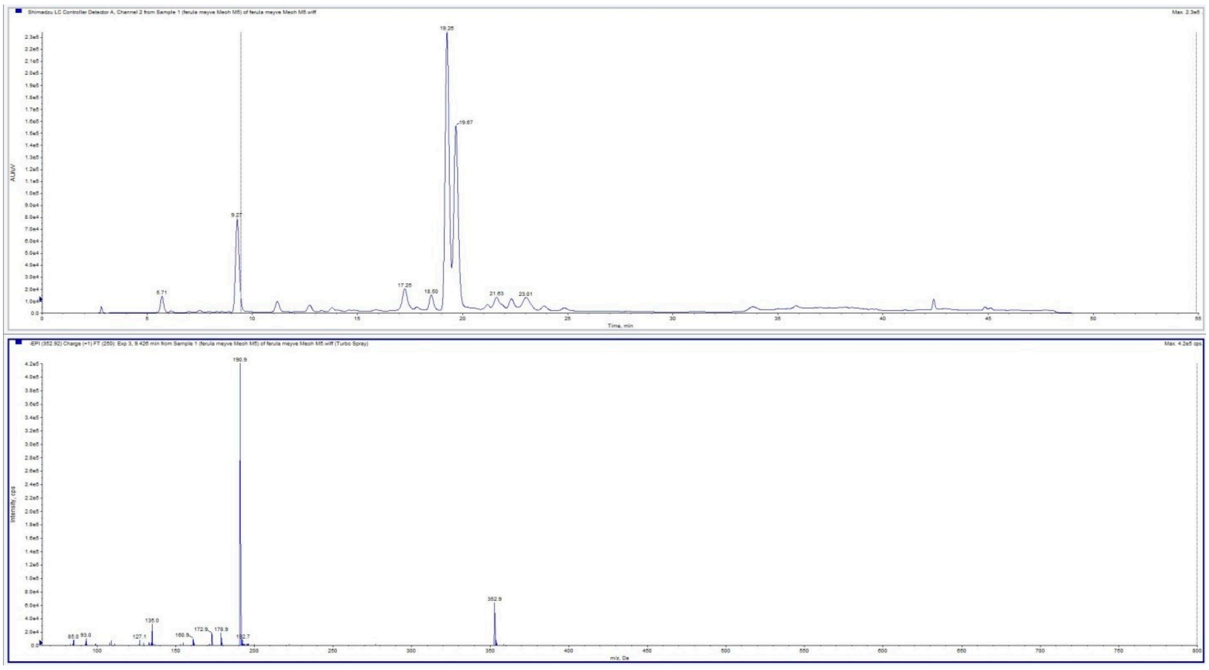

Figure. S2. LC-MS/MS spectrum of 5-caffeoylquinic acid (**13**, chlorogenic acid)

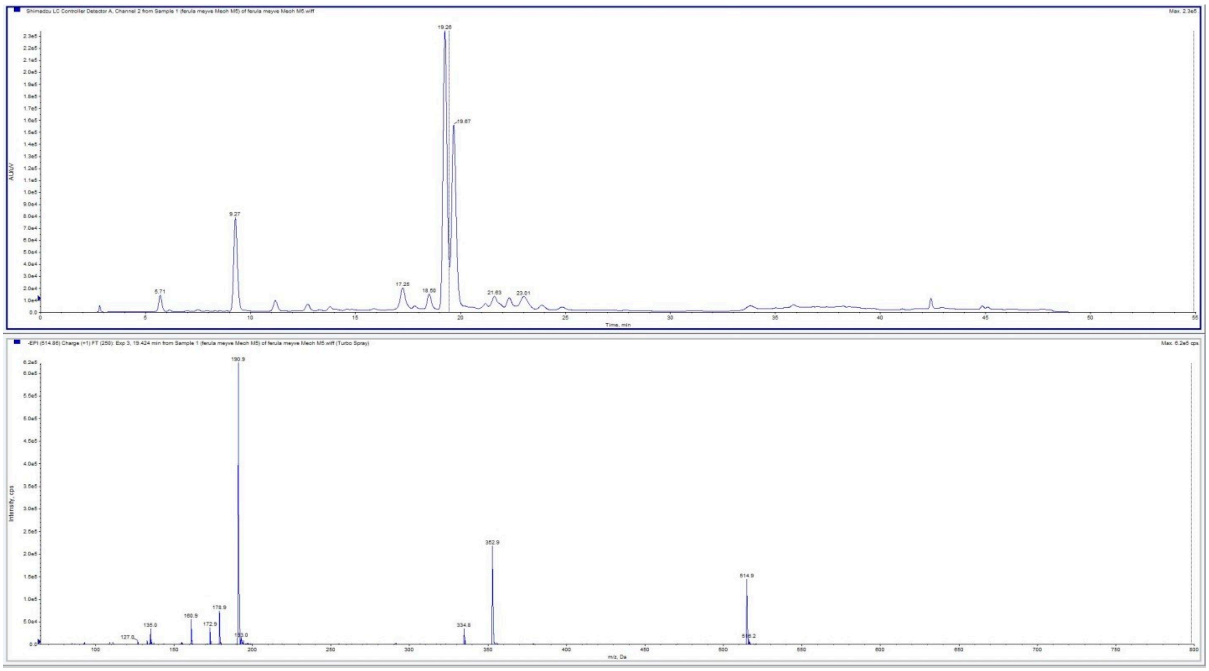

Figure. S3. LC-MS/MS spectrum of 1,5-dicaffeoylquinic acid (**16**, cynarine)

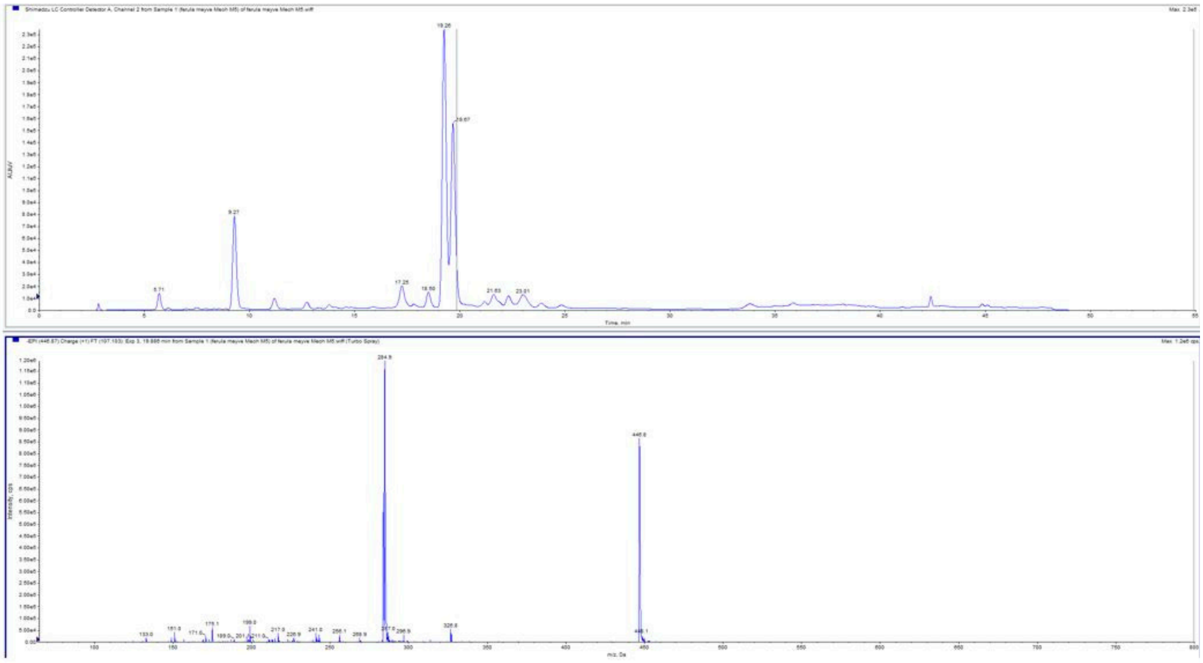

Figure. S4. LC-MS/MS spectrum of Luteolin 7-glucoside (**18**, cynaroside)
